# Perioperative treatment in resectable gastric cancer with spartalizumab in combination with fluorouracil, leucovorin, oxaliplatin and docetaxel (FLOT): a phase II study (GASPAR)

**DOI:** 10.1186/s12885-022-09623-z

**Published:** 2022-05-12

**Authors:** Mélanie Dos Santos, Justine Lequesne, Alexandra Leconte, Stéphane Corbinais, Aurélie Parzy, Jean-Marc Guilloit, Sharmini Varatharajah, Pierre-Emmanuel Brachet, Marine Dorbeau, Dominique Vaur, Louis-Bastien Weiswald, Laurent Poulain, Corentin Le Gallic, Marie Castera-Tellier, Marie-Pierre Galais, Bénédicte Clarisse

**Affiliations:** 1 Clinical Research Department, UNICANCER, Centre François Baclesse, 3 Avenue du Général Harris, 14000 Caen, France; 2 Department of Medical Oncology, UNICANCER, Centre François Baclesse, 3 Avenue du Général Harris, 14000 Caen, France; 3 Department of Surgery, UNICANCERCentre François Baclesse, 14000 Caen, France; 4 Department of Pathology, UNICANCERCentre François Baclesse, 14000 Caen, France; 5 Department of Cancer Biology and Genetics, UNICANCERCentre François Baclesse, 14000 Caen, France; 6 UNICANCER, Centre François Baclesse, 14000 Caen, France; 7 Normandie University, UNICAEN, ANTICIPE, ORGAPRED Platform, Caen, France

**Keywords:** Gastric cancer, Gastroesophageal junction cancer, Neoadjuvant treatment, Immunotherapy, Spartalizumab

## Abstract

**Background:**

Perioperative chemotherapy and surgery are a standard of care for patients with resectable gastric or gastroesophageal junction (GEJ) adenocarcinoma. However, the prognosis remains poor for this population. The FLOT (fluorouracil, leucovorin, oxaliplatin, and docetaxel) regimen is considered as the new standard chemotherapy regimen for perioperative strategy, despite associated with a 5-year overall survival rate (OS) amounting 45% following radical surgery.

Immunotherapy with antibodies that inhibit PD-1/ PD-L1 interaction has recently emerged as a new treatment option with promising and encouraging early trial results for patients with advanced or metastatic gastric or GEJ adenocarcinoma. Currently, no trials have investigated the impact of perioperative immunotherapy in combination with chemotherapy for resectable gastric or GEJ adenocarcinoma.

**Methods:**

GASPAR trial is a multicenter open-label, nonrandomized, phase II trial to evaluate the efficacy and safety of Spartalizumab in combination with the FLOT regimen as perioperative treatment for resectable gastric or GEJ adenocarcinoma. The main endpoint is the proportion of patients with pathological complete regression (pCR) in the primary tumour after preoperative treatment.

Systemic treatment will include a pre-operative neoadjuvant and a post-operative adjuvant treatment, during which FLOT regimen will be administered every two weeks for 4 cycles and Spartalizumab every four weeks for 2 cycles.

For patients with confirmed tumor resectability on imaging assessment, surgery will be realized within 4–6 weeks after the last dose of preoperative chemotherapy. Post-operative systemic treatment will then be initiated within 4–10 weeks after surgery.

Using a Simon’s two-stage design, up to 67 patients will be enrolled, including 23 in the first stage.

**Discussion:**

Currently, no trials have investigated the impact of immunotherapy in combination with FLOT chemotherapy as perioperative treatment for resectable gastric or GEJ adenocarcinoma. Some studies have suggested a change in the tumor immune micro-environment following neoadjuvant chemotherapy in this setting, reinforcing the relevance to propose a phase II trial evaluating efficacy and safety of Spartalizumab in combination with perioperative chemotherapy, with the aim of improving treatment efficacy and survival outcomes.

**Trial registration:**

NCT04736485, registered February, 3, 2021.

## Background

Gastric cancer represents the fifth most common cancer and the third leading cause of cancer deaths in the world [1]. Perioperative chemotherapy and surgery are a standard of care for patients with resectable gastric or GastroEsophageal Junction (GEJ) adenocarcinoma. Despite this combination of treatment, the prognosis remains poor for this population.

Perioperative treatment was considered as the standard compared to surgery alone according to the randomized MAGIC trial, evaluating ECF (Epirubicin, Cisplatin and Fluorouracile), 3 pre- and 3 post-operative cycles in 503 patients with resectable locally advanced gastric or GEJ adenorcarcinoma [2]. Especially, an improved Overall Survival (OS) with a 5-year survival rate of 36% was observed, *versus* 23% for surgery alone. However, in a controlled open-label phase II/III trial conducted among 716 patients, the FLOT regimen (Fluorouracil, Leucovorin, Oxaliplatin, and doceTaxel), 4 pre- and 4 post-operative cycles, was associated with better OS compared to ECF as perioperative chemotherapy, with 50 months *versus* 35 months in median [3]. Moreover, there was a higher proportion of pathological Complete Response (pCR): 16% [95% CI: 10–23] versus 6% [95% CI: 3–11] [4]. Furthermore, a recent meta-analysis showed that pCR was clearly associated with lower risk of death and recurrence compared with patients with any residual disease, among 1 143 patients with resectable gastric or GEJ cancer, after neoadjuvant chemotherapy and radical surgery [5]. The FLOT regimen thus appears as the new standard chemotherapy regimen for perioperative strategy of resectable gastric or GEJ adenocarcinoma. However, the 5-year OS rate remains only at 45% following radical surgery [3]. New approaches are needed to improve these outcomes.

PD-1 is a critical immune checkpoint receptor. It acts through its ligands, PD-L1 and PD-L2, while transducing a signal that inhibits T-cell proliferation, cytokine production, and cytolytic function, attenuating tumor immunity and facilitating tumor progression [6, 7]. PD-1 and its ligand PD-L1 are expressed on up to 50% of gastric or GEJ tumors, with a controversial impact on survival [8, 9]. Immunotherapy with antibodies that inhibit PD-1/PD-L1 interaction has recently emerged as a new treatment option with promising and encouraging early trial results for patients with advanced or metastatic gastric or GEJ adenocarcinoma [10]. Muro et al. conducted a phase Ib trial (KEYNOTE-012) in 39 patients with PD-L1-positive advanced gastric or GEJ adenocarcinoma to investigate the safety and activity of the anti-PD-1 antibody pembrolizumab [11]. Pembrolizumab demonstrated a 22% objective response rate with a manageable toxicity profile. Fuchs et al. conducted the KEYNOTE-059 phase II trial with pembrolizumab monotherapy in 259 patients with previously treated advanced gastric and GEJ cancer (at least 2 lines of treatment) and showed durable responses, with great response rate, especially for PD-L1 positive tumors [12]. Results from these studies allowed approval of pembrolizumab by the US FDA as third line treatment for patients with advanced or metastatic gastric or GEJ cancer PD-L1–positive. Moreover, an update of the KEYNOTE-059 trial demonstrated a manageable safety and promising efficacy of first-line immunotherapy combined with chemotherapy (cisplatin and fluorouracil) [13]. In a first-line study (KEYNOTE-062), Shitara et al. showed encouraging benefit with pembrolizumab versus chemotherapy among patients with untreated advanced gastric and GEJ cancer with higher levels of PD-L1 and microsatellite instability–high (MSI-H) tumours [14]. The CheckMate 649 trial recently showed that nivolumab and chemotherapy versus chemotherapy alone improved OS and progression-free survival (PFS) as first-line treatment in patients with non-HER-2-positive advanced gastric, GEJ or oesophageal cancer [15].

PDR001 (Spartalizumab) is a high-affinity, ligand-blocking, humanized Immunoglobulin G4 monoclonal antibody directed against PD-1 that blocks the binding of PD-L1 and PD-L2 [16]. Spartalizumab has demonstrated pharmacodynamic activity and a favorable toxicology profile in preclinical studies [17]. The available safety data from clinical studies indicate that PDR001 is generally well tolerated. As of the safety cut-off date of 26-Mar-2020, 1 702 patients across the 17 Novartis-sponsored clinical studies described have been treated with PDR001. In the open label multicenter phase I/II study of the safety and efficacy of PDR001 administered to patients with advanced malignancies, the results of phase I dose escalation among 58 patients have been published [17]. The maximum tolerated dose was not reached. The recommended phase II doses were determined as 400 mg Q4W or 300 mg Q3W. No dose-limiting toxicities were observed, and adverse events included those typical of other PD-1 antibodies. In the phase I step of this trial [17], the most common treatment-related adverse events of any grade were fatigue (22%), diarrhea (17%), pruritus (14%), hypothyroidism (10%), and nausea (10%).

**Fig. 1:**
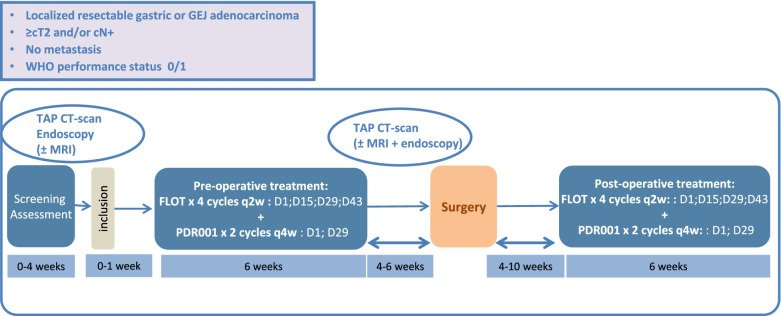
Flow chart of GASPAR study

In the GASPAR study herein presented, we aim to evaluate the efficacy and safety of Spartalizumab in combination with the FLOT regimen as perioperative treatment for resectable gastric or GEJ adenocarcinoma. Ancillary biological exploration is also planned to identify subgroups of responder patients. Indeed, cancer immunotherapies represent a major novel class of antitumor agents. However, the mechanism of action of these exciting new therapies is not completely understood and much remains to be learned regarding how best to leverage these new drugs in treating patients. Thus, to aid future patients, it is important to investigate the determinants of response or resistance to cancer immunotherapy and other treatments administered. These efforts may identify predictive biomarkers and generate information that could help in patient selection with personalized medicine programs (Fig. 1).

## Methods / design

The GASPAR study is a multicenter, open-label, non-randomized phase II trial conducted to evaluate the efficacy and safety of Spartalizumab in combination with the FLOT regimen as perioperative treatment for resectable gastric or GEJ adenocarcinoma in patients (Fig. 1). The GASPAR protocol and this manuscript have been written in accordance with standard protocol items, namely recommendations for interventional trials (SPIRIT).

### Primary outcome

The primary objective of the study is to assess the pathologic response after pre-operative treatment by Spartalizumab in combination with the FLOT regimen for resectable gastric or GEJ adenocarcinoma.

### Secondary outcomes

The secondary objectives are:

- To evaluate the impact of perioperative treatment on survival outcomes (disease-free and overall survivals)
- To evaluate the histological R0 resection margin
- To establish the association between pCR and survival outcomes (disease-free and overall survivals)
- To determine the safety profile of the combination of Spartalizumab and FLOT regimen
- To evaluate the post-operative morbidity and mortality

### Study population

Eligibility criteria are precised in Table 1. The GASPAR study addresses patients with untreated localized gastric or GEJ adenocarcinoma considered resectable.

**Table 1:** Eligibility criteria

Inclusion criteria	Non-inclusion criteria
● Patient ≥ 18 years at the day of consenting to the study ● Provision of informed consent prior to any study specific procedures ● Untreated localized gastric or GEJ adenocarcinoma considered resectable (clinical stage ≥ cT2 and/or cN + and no metastasis) ● Histologically confirmed adenocarcinoma ● ECOG performance status score ≤ 1 ● Tumor tissue must be provided for biomarker analyses (fresh or archival with an FFPE tissue block) ● All subjects must consent to allow the acquisition of blood samples for performance of correlative studies ● Screening laboratory values must meet the following criteria: WBC ≥ 2000/ mm^3^, Neutrophils ≥ 1500/ mm^3^, Platelets ≥ 100 000/ mm^3^, Hemoglobin ≥ 9.0 g/dL, Bilirubin ≤ 1.5 × ULN, AST and ALT ≤ 3 × ULN, measured or calculated creatinine ≥ 50 ml/min clearance (CrCl) (using the Cockcroft-Gault formula), Potassium ≥ LLN, Magnesium ≥ LLN and Calcium ≥ LLN ● Female subject of childbearing potential must have a negative urine or serum pregnancy test within 72 h before study start ● Subject in reproductive age must be willing to use adequate contraception during the study and at least 9 months in men and 12 months in women after the last dose of investigational drug. In addition, given the toxicities observed on the male reproductive system, a conservation of gametes will be proposed for men ● Subject affiliated to a social security regimen	● Subject with any distant metastasis ● Subject with no recovering from the effects of major surgery or significant traumatic injury within 14 days before inclusion ● Documented significant cardiovascular disease within the past 6 months before the first dose of study treatment, including: history of congestive heart failure (defined as NYHA III or IV), myocardial infarction, unstable angina, coronary angioplasty, coronary stenting, coronary artery bypass graft, cerebrovascular accident or hypertensive crisis ● History of anterior organ transplant ● Pneumonitis or interstitial lung disease ● History of other malignancy within the previous 3 years (except for appropriately treated in-situ cervix carcinoma and non-melanoma skin carcinoma) ● Active, known, or suspected autoimmune disease ● Subject with a condition requiring systemic treatment with either corticosteroids (> 10 mg daily prednisone equivalent) or other immunosuppressive medications within 14 days of start of study treatment ● Known history of HIV or HBV infection, history of active tuberculosis, active HCV infection ● Vaccination with live vaccine within 30 days before the first dose of study treatment ● Prior treatment with an anti-PD-1, anti-PD-L1, anti-PD-L2 or any other antibody or drug specifically targeting T-cell co-stimulation or checkpoint pathways ● Recent or concomitant treatment with brivudine ● Prior anticancer therapy for the current malignancy ● Known hypersensitivity to any of the study drugs or their excipients ● Chronic inflammable gastro-intestinal disease ● Uracilemia ≥ 16 ng/ml ● QT/QTc > 450 ms for men and > 470 ms for women ● Peripheral neuropathy ≥ Grade II ● Uncontrolled diabetes ● Active infection requiring systemic therapy ● Participation in another therapeutic clinical study ● Patient deprived of liberty or placed under the authority of a tutor ● Patient assessed by the investigator to be unable or unwilling to comply with the requirements of the protocol

### Study sites

The list of study sites is indicated on https://clinicaltrials.gov/ct2/show/NCT04736485. The participation of 13 French centres is planned (Table 2).

**Table 2:** Participating centers

**INVESTIGATORS**	**PARTICIPATING FRENCH COMPREHENSIVE CANCER CENTRES**
**Coordinating investigator:** Dr Mélanie DOS SANTOS **Co-investigators:** Dr Marie-Pierre GALAIS Dr Stéphane CORBINAIS Dr Aurélie PARZY Dr Pierre-Emmanuel BRACHET Dr Georges EMILE Dr Emeline MERIAUX	**Centre François Baclesse, CAEN**
**Main investigator:** **Pr Thomas APARICIO** **Co-investigators:** Dr Jean-Marc GORNET Dr Nelson LOURENCO Dr Nassim HAMMOUDI Dr Nicolas ASESIO Dr Delphine SALFATI	**Assistance Publique – Hôpitaux de Paris (AP-HP), Hôpital Saint-Louis, PARIS**
**Main investigator:** Dr Romain Desgrippes **Co-investigators:** Dr Anaïs BODERE	**Centre Hospitalier, SAINT-MALO**
**Main investigator:** Pr Christophe BORG **Co-investigators:** Dr Marine JARY Dr Francine FEIN Dr Thierry NGUYEN Dr Hamadi ALMOTLAK Dr Angélique VIENOT Dr Elodie KLAJER	**University Hospital, BESANCON**
**Main investigator:** Dr Sandrine HIRET **Co-investigators:** Dr Ludovic DOUCET Dr Camille MOREAU BACHELARD Dr Judith RAIMBOURG Dr Hélène SENELLART Dr Amélie MALLET Dr Frédéric DUMONT	**Institut de Cancérologie de l’Ouest, site NANTES**
**Main investigator:** Dr Guillaume PIESSEN **Co-investigators:** Dr Anthony TURPIN Dr Anne PLOQUIN Dr Christophe DESAUW Dr Nicolas BERTRAND Dr Anne GANDON Dr Clément DUBOIS	**Regional University Hospital, LILLE**
**Main investigator:** Dr Emilie SOULARUE **Co-investigators:** Dr Christophe LOUVET Dr Mostefa BENNAMOUN Dr Marie-Liesse JOULIA	**Institut Mutualiste Montsouris, PARIS**
**Main investigator:** Dr Mathilde BRASSEUR **Co-investigators:** Pr Olivier BOUCHE Dr Damien BOTSEN	**University Hospital Robert Debré, REIMS**
**Main investigator:** Dr Samuel LE SOURD **Co-investigators:** Dr Héloïse BOURIEN Dr Alexandra FRELAU Dr Florian ESTRADE Dr Thomas GRAINVILLE Dr Céline LESCURE Dr Astrid LIEVRE Dr Léa MUZELLEC Dr Eugénie RIGAULT Dr Claude BERTRAND	**Centre Eugène Marquis, RENNES**
**Main investigator:** Dr Laetitia DAHAN **Co-investigators:** Dr Muriel Duluc Dr Emmanuelle NORGUET- MONNEREAU Dr Catherine FONTAINE Dr Maelle RONY	**Assisantce Publique – Hôpitaux de Marseille, MARSEILLE**
**Main investigator:** Dr Emmanuelle SAMALIN **Co-investigators:** Dr Marc YCHOU Dr Antoine ADENIS Dr Thibault MAZARD Dr Fabienne PORTALES Dr Marie-Cécile BORNE-GERLOTTO Dr Dalila FERROUKHI Dr Blandine GALLET-SUCHET Dr Alex KOUAME Dr Stéphane POUDEROUX Dr Marie ALEXANDRE Dr Marie VINCHES	**Institut Régional du Cancer, MONTPELLIER**
**Main investigator:** Dr Rosine GUIMBAUD **Co-investigators:** Dr Corinne COUTEAU Dr Marion DESLANDRES Dr Nadim FARES Dr Marion JAFFRELOT Dr Pascale RIVERA Dr Isabelle ROQUE	**University Hospital, TOULOUSE**
**Main investigator:** Dr Simon PERNOT **Co-investigators:** Dr Dominique BECHADE Dr Marianne FONCK	**Institut Bergonié, BORDEAUX**

### Study treatments

Eligible patients who have completed screening and have signed the written informed consent to participate to this phase II trial will receive a treatment by Spartalizumab plus FLOT regimen, initiated within 15 days after inclusion. Systemic treatment will include a pre-operative neoadjuvant 8-week phase of treatment and a post-operative 8-week phase of treatment.

The administered treatment will be FLOT associated to Spartalizumab as follows:

Standard FLOT regimen:

o Docetaxel 50 mg/m^2^ IV infusion on D1
p Oxaliplatine 85 mg/m^2^ IV infusion on D1
q Leucovorin 200 mg/m^2^ IV infusion on D1
r Fluorouracile 2600 mg/m^2^ 24 h IV infusion on D1

Chemotherapy will be administered every two weeks for 4 pre-operative cycles (8 weeks) and 4 post-operative cycles (8 weeks).

Spartalizumab (PDR001): patients will receive the fixed dose of 400 mg per IV infusion over 30 minutes on D1 every four weeks for 2 pre-operative cycles (8 weeks) and 2 post-operative cycles (8 weeks).

For patients with confirmed resectability of the tumor by an imaging assessment (TAP CT-scan and optional MRI and endoscopy), surgery will be realized within 4–6 weeks after the last dose of preoperative chemotherapy and will depend on tumoral localization:

for gastric tumors, surgery will consist on a total or subtotal distal (for antropyloric tumors) gastrectomy with D2 lymphadenectomy,

for type 1 GEJ tumors, transthoracic esophagectomy (Ivor-Lewis procedure) with resection of the proximal stomach and 2-field (mediastinal and abdominal) lymphadenectomy,

for type 2 or 3 GEJ tumors, gastrectomy with transhiatal distal oesophagectomy and D2 lymphadenectomy.

Local pathologists from selected expert centers will perform standardized evaluation of pathological response in surgically resected specimens. Tumour regression grade will be assessed according to the Becker regression criteria [18].

Post-operative systemic treatment will be initiated within 4–10 weeks after surgery.

Premedication is not recommended for Spartalizumab. Also, it is recommended to use antiemetic treatment according to the ASCO/MASCC recommendation guideline with aprepitant and setron before FLOT regimen.

To anticipate some potential interactions with Spartalizumab and/or FLOT regimen, the use of concomitant medications is defined in the protocol. Thus, some treatments and/or procedures are permitted, namely G-CSF (in secondary prophylaxis of severe or febrile neutropenia, or in primary prophylaxis from first cycle of treatment), erythropoietin and/or transfusions, anti-diarrheal medications in case of diarrhea, pain medication, and/or nutritional support, at the discretion of the investigator. Conversely, other systemic anticancer agents (chemotherapy, hormonal therapy other than megestrol acetate, immunotherapy) or other treatments not part of protocol-specified anticancer therapy, live vaccines, systemic glucocorticoids for any purpose other than to modulate symptoms from an adverse event that is suspected to have an immunologic etiology (the use of physiologic doses of corticosteroids may be approved after consultation with the Sponsor) are not authorized. In addition, drugs known to prolong the QTc interval should be used with caution.

### Study assessments

The overview of study assessments and procedures is detailed in Table 3. Tumoral evaluation will be performed with CT-scan (thoracic and abdomino-pelvic) at baseline, before surgery, at the end of treatment, thereafter every 3 months for the first 2 years and every 6 months for the next 3 years in the absence of tumoral progression. Disease assessment evaluation will be determined locally according to RECIST v1.1 (Response Evaluation Criteria in solid Tumors) criteria.

**Table 3:** Overview of study assessments of the GASPAR trial

	**Before inclusion** (within 28 days prior inclusion)	**DURING TREATMENT**									**End of treatment** 30 days after the end of treatment (± 7 days)	**Follow-up** every 3 months up to progression	**Overall survival** after disease progression
		**Pre-operative treatment **^**4**^				**Before SURGERY**	**Post-operative treatment **^**5**^						
		**D1**	**D15**	**D29**	**D43**	Within 3 weeks before surgery	**D1**	**D15**	**D29**	**D43**			
**FLOT** *(q2w)* **Spartalizumab PDR001** *(q4w)*		♦ ♦	♦	♦ ♦	♦		♦ ♦	♦	♦ ♦	♦			No study visit is required. The following treatment/examns are at the discretion of physician
**Signed Informed Consent** *before any study procedures*	✔												
**Clinical assessment** Physical examination including weight, ECOG, vital signs Adverse Events collection and concomitant treatments	✔	✔ ✔	✔ ✔	✔ ✔	✔ ✔	✔ ✔	✔ ✔	✔ ✔	✔ ✔	✔ ✔	✔ ✔	✔	
**Biological assessment** Hematology and biochemistry^**1**^	✔	✔^**2,3**^	✔^**3**^	✔^**3**^	✔^**3**^	✔	✔^**3**^	✔^**3**^	✔^**3**^	✔^**3**^	✔		
Uracilemia	✔												
Urine or serum pregnancy test		✔^**3**^		✔^**3**^			✔^**3**^		✔^**3**^				
Thyroid-function: TSH, free T4	✔	✔^**2**^		✔^**3**^		✔	✔^**3**^		✔^**3**^		✔		
Tumor markers: CEA, CA 19.9	✔	✔^**2**^				✔	✔^**3**^				✔		
**ECG**	✔	✔^**7**^	✔^**7**^	✔^**7**^	✔^**7**^		✔^**7**^	✔^**7**^	✔^**7**^	✔^**7**^			
**CT-scan** (thoracic and abdomino-pelvic) **MRI optional**	✔ ✔					✔ ✔					✔ ✔	✔^**8**^ ✔	
**Endoscopy**	✔^**9**^					✔ optional							
**Blood samples for translational research**^**10**^	✔					✔	✔				✔	✔^**6**^	

^1^ CBC-platelets, Creatininemia, kaliema, magnesemia, calcemia, albumin, glycemia, lipase, bilirubin, ALT, AST, GGT

^2^ Only if realized more 3 days before D1

^3^ Within 3 days before treatment administration

^4^ The initial combination treatment by FLOT regimen plus Spartalizumab should be initiated within 7 days after inclusion

^5^ The combination treatment by FLOT regimen plus Spartalizumab should be initiated within 4–10 weeks after surgery

^6^ Blood sample only for follow-up at 3 months

^7^ To be realized before and after oxaliplatin intravenous infusion

^8^ Every 3 months for the first 2 years and every 6 months for the next 3 years

^9^ Within 6 weeks prior to inclusion with available archival tumor (otherwise, fresh tumor) And optional fresh tumor biopsies only at François Baclesse site for organoids research (additional specific consent form)

^10^ Mandatory blood samples for ctDNA

And optional PBMC at baseline only at François Baclesse site for organoids research (additional specific consent form)

For further ancillary biological studies, we plan to constitute a blood and tumor collection with the aim to evaluate the impact of biomarkers (ctDNA and tissue) in terms of oncological outcomes and response to treatment. These biomarkers may include ctDNA levels over time, PD-L1 expression, MSI status, EBV (Epstein-Barr Virus) status and TMB (Tumor Mutational Burden). Exploration of tumor organoid culture is also planned only for patients enrolled in Centre François Baclesse with additional specific written consent form.

### Statistical design overview

#### Sample size determination

We used an optimal Simon’s two stage phase II design to estimate the sample size. According to Al-Batran S-E et al. outcomes [4], patients rate achieving pathological complete regression with FLOT regimen was 16% [95%CI: 10–23] (observed in 128 patients). To assess efficacy of FLOT regimen combined with Spartalizumab, we assume a histological complete response rate *p* < 10% as unacceptable (corresponding to the lower CI limit of response rate in Al-Batran), and a response rate *p* > 23% as demonstrating efficacy of the treatment combination (corresponding to the upper CI limit). With an alpha level of 5% and statistical power of 80%, 58 assessable patients are required, including 20 in the first stage.

Taking into account that 15% of patients will be lost or non-assessable, we plan to include a total of 67 patients in the trial (23 in the first stage and 44 in the second).

#### Statistical analyses

All enrolled patients who will receive at least one dose of study medication will be evaluable for the efficacy analysis, as well as included in the safety analysis. A safety analysis will be planned after a follow-up of one month following surgery of the tenth enrolled patient, during which enrolment will be halted. It will aim to precisely describe safety data up to one month post-surgery, the potential delays in performing surgery and the reasons for postponing it, as well as the perioperative complications that will have occurred.

After the 23th inclusion, the planned interim analysis on efficacy will be performed, during which enrolment will not be halted. According to Simon’s two-stage design, a minimum of 3 complete responses out of 20 assessable patients in the first stage will allow pursuing the study in the second stage. Otherwise, the study will stop for insufficient efficacy.

On the 58 assessable patients considered for the final analysis, the study will conclude to efficacy if at least 10 complete responses are observed. Additionally, the response rate will be estimated with a 95%CI confidence interval.

Then, Chi-squared test and T-Student test will be used to measure association of, respectively, qualitative and quantitative variables with pCR response. Time-to-event variables will be summarized by the Kaplan–Meier estimator. Association of time-to-event variables with factors of interest will be measured by a Cox model and the log-rank test. Adverse events of the treatment combing Spartalizumab and FLOT regimen will be described according to NCI CTCAE criteria v5.0 and tabulated by grade and frequencies, based on time of occurrence and relationship to treatment. The tolerance profile of the association will be summarized by duration of treatment, reasons of discontinuation, dose reduction rates and reasons for dose reductions.

### Data monitoring committee

An Independent Data Monitoring Committee (IDMC) will be set-up to ensure the protection of patients, to ensure the ethical conduct of the study, to evaluate the benefit/risk ratio of the study and to ensure an independent review of the scientific outcomes during and at completion of the study.

The committee will include a biostatistician, a pharmacologist and a medical oncologist.

The members of the IDMC will be consulted before the trial initiation, at safety analysis one month after surgery of the 10th enrolled patients, thereafter, at the interim and final analyses.

### Data management

A Web Based Data Capture (WBDC) system will be used for data collection and query handling. The investigator will ensure that data are recorded on the eCRFs as specified in the study protocol and in accordance with the instructions provided.

The investigator ensures the accuracy, completeness, and timeliness of the data recorded and of the provision of answers to data queries according to the Clinical Study Agreement. The investigator will sign the completed eCRFs. A copy of the completed eCRFs will be archived at the study site.

## Withdrawal from study

The reasons for why a patient may discontinue to participate to the study or interrupt the treatment include the following circumstances:

- Disease progression
- Need to initiate another anti-tumor treatment, including radiotherapy
- Unacceptable toxicity, not compatible with study treatment
- Patient's decision (the data already collected during the search can be kept and exploited unless the patient opposes it)
- Intercurrent illness or other reason that requires stopping treatment of the study
- Patient lost to view
- Investigator's decision.

Any patient who prematurely withdraws from the study treatment only will continue to be followed, unless he/she withdraws from the study.

## Discussion

Currently, no trials have investigated the impact of neoadjuvant immunotherapy in combination with chemotherapy for resectable gastric or GEJ adenocarcinoma. Nevertheless, some studies suggest a change in the tumor immune micro-environment following neoadjuvant chemotherapy in locally advanced gastric cancer, with an increased expression of immune markers. Thus; tumor samples, before and after neoadjuvant chemotherapy, of 60 patients with locally advanced gastric cancer were retrospectively identified and analyzed by multiplex immunohistochemistry, with a panel including PD-1 and PD-L1 [19]: following neoadjuvant chemotherapy, the overall median expression levels of PD1 and PD-L1 were significantly increased; moreover, high upregulation levels of these checkpoint molecules were correlated with survival benefits.

All these data are encouraging in the use of immunotherapy in combination with perioperative chemotherapy, with the aim of improving treatment efficacy and survival outcomes. In this context, we propose a non-randomized phase 2 study to assess the efficacy and safety of Spartalizumab in combination with the FLOT regimen as perioperative treatment for resectable gastric or GEJ adenocarcinoma.

## Data Availability

Not applicable.

## Abbreviations

AE: Adverse event
ALT: Alanine aminotransferase
ASCO: American Society of Clinical Oncology
AST: Aspartate aminotransferase
CA 19.9: Carbohydrate antigen 19.9
CBC: Complete blood cells count
CEA: Carcinoembryonic antigen
CI: Confidence intervals
CT: Computed tomography
CTCAE: Common Terminology Criteria for Adverse Events
ctDNA: Circulating tumor DNA
EBV: Epstein–barr virus
ECG: Electrocardiogram
ECOG: Eastern Cooperative Oncology Group
eCRF: Electronic case report form
FDA: Food and Drug Administration
FFPE: Formalin-fixed paraffin-embedded
GEJ: Gastroesophageal junction
GGT: Gamma glutamyltransferase
HBV: Hepatitis B virus
HCV: Hepatitis C virus
HIV: Human immunodeficiency virus
IDMC: Independent data monitoring committee
MASCC: Multinational Association of Supportive Care in Cancer
MRI: Magnetic resonance imaging
MSI: Microsatellits instability
NYHA: New York Heart Association
NCI: National cancer institute
OS: Overall survival
PBMC: Peripheral blood mononuclear cells
pCR: Pathological complete regression
PD-1: Programmed death-1
PD-L1: Programmed death-ligand 1
PD-L2: Programmed death-ligand 2
QTc: Corrected QT interval
RECIST: Response evaluation criteria in solid tumors
SPIRIT: Standard Protocol Items, Recommendations for Interventional Trials
T4: Serum thyroxine
TAP: Thorax, abdomen and pelvis
TMB: Tumor mutational burden
TSH: Thyroid stimulating hormone
WBC: White blood cell count
WBDC: Web Based Data Capture
